# Predicting the defensive performance of individual players in one vs. one soccer games

**DOI:** 10.1371/journal.pone.0209822

**Published:** 2018-12-31

**Authors:** Robbie S. Wilson, Nicholas M. A. Smith, Paulo Roberto Pereira Santiago, Thiago Camata, Solange de Paula Ramos, Fabio Giuliano Caetano, Sergio Augusto Cunha, Ana Paula Sandes de Souza, Felipe Arruda Moura

**Affiliations:** 1 School of Biological Sciences, The University of Queensland, Brisbane, Queensland, Australia; 2 School of Life and Environmental Sciences, The University of Sydney, Sydney, New South Wales, Australia; 3 School of Physical Education and Sports of Ribeirão Preto, University of Sao Paulo, Ribeirão Preto, Brazil; 4 Sport Sciences Department, State University of Londrina, Londrina, Brazil; 5 College of Physical Education, University of Campinas, Campinas, Brazil; 6 University of Brasilia (UnB), Brasilia, Brazil; Nottingham Trent University, UNITED KINGDOM

## Abstract

The aim of this study was to use technical skill and physical performance and coaches’ rankings to predict the defensive performance of junior soccer players. Twenty-one male players (mean age 17.2 years, SD = 1.1) were recruited from the Londrina Junior Team Football Academy in Brazil. Data were collected during regular training sessions. After participants had warmed up, players were asked to either dribble the ball or sprint through five custom circuits that varied in average curvature (0–1.37 radians.m^-1^). In addition, four coaches were asked to rank the players from best to worst in defensive ability. Dribbling, sprinting, and coaches’ rankings were then compared with defending performance as assessed in the one vs. one competitions (N = 1090 paired-trials: 40–65 trials per individual), in which they acted as defender or attacker in turn. When defending, the objective was to steal the ball or prevent the attacker from running around them with the ball into a scoring zone. Testing occurred over three days. Overall, dribbling performance (r = 0.56; P = 0.008) and coaches’ ranking (r = 0.59; P = 0.004) were significantly related to defensive ability; sprinting performance was not (r = 0.20; P = 0.38). Though dribbling performance and coaches’ ranking each explained 30% and 37% of the variance in defensive performance, respectively, the two predictors were not related (r = 0.27; P = 0.23), so combined these traits explained more than half the variance in defensive performance. In conclusion, the current study demonstrates that including only one metric of closed-skill performance—dribbling speed—doubles the ability of coaches to identify their best defensive players in one vs. one scenarios.

## Introduction

Sports scientists aim to identify the physiological, morphological, sociological, psychological, and technical traits associated with success in elite sports [1–5]. By showing what factors predict success, scientists can improve coaching, training, and development of youth and professional players [4]. In some sports, like athletics, swimming and rowing, success can be attributed to a few easily measured traits like power, speed, and endurance [6–8]. However, many team sports, such as soccer, rugby, and basketball, are complex and interactive, and athletic traits like power, speed, and endurance are not sufficient to identify talented youth players [1–3, 9–13]. In addition, success in team sports is likely to come in many forms, via different combinations of athletic, skill, and psychological traits [3, 5, 9–14]. In soccer, for example, there is not likely to be a single formula to predict success. Short and agile players with high technical ability could possibly achieve equal success to tall, powerful players with lower agility and ball-handling skills, although probably in different positions (e.g. central defenders vs wide defenders in soccer). Currently, most protocols to identify talent in team sports do not account for variation in ability at an individual level. Instead, success is generally predicted on those traits that can discriminate between elite and non-elite levels [3, 15–17]. In soccer, traits such as dribbling speed, passing and shooting accuracy, heading ability, and wall-volley accuracy are all highly repeatable and can often reliably discriminate among players in elite and non-elite levels [3, 9, 11, 12, 18–21]. But because these studies do not quantify variation in performances among individuals, they cannot differentiate among players within each playing-level and identify which combination of traits best predict success [13, 22].

To predict which elite players are most likely to succeed and identify the different combinations of traits that will lead to elite success, variation in traits should be analysed among individuals and related to their performances in matches or in match-realistic tasks. To test this idea, Wilson and colleagues [13] measured aspects of skill, balance, and athleticism in soccer players before testing their success in 11-a-side matches. In their study, soccer-specific motor skill—rather than athletic ability—best predicted match success. By quantifying the importance of motor skill to soccer, Wilson and colleagues [13] took an important step forward in talent identification, but the metrics of success used in their study were based on overall match play and did not account for differences in traits that would be associated with specific team positions. Many sports, like soccer, require numerous player types to perform different activities (e.g. defender vs striker), which are likely to require different sets of traits for success [16].

If one could predict the success of players in specific roles in matches (e.g. central defenders, wide defenders, attackers), coaches could design unique training regimes for individual players and player-types. Identifying how different skill, athletic and morphological traits combine to affect success in specific tasks within matches may also provide a better understanding of how certain combinations of traits lead to similar levels of success. Defense is fundamental to soccer play, and coaches would benefit from identifying individual players that excel at: (i) preventing opposition teams from reaching threatening positions on the field, (ii) clearing the ball when it reaches threatening positions, (iii) avoiding being beaten by attackers with the ball when isolated one vs. one, and (iv) winning or stealing the ball from opponents. Within professional leagues, a player’s defensive ability can be estimated during matches, but a closed-skill assessment would improve the identification of talented youth. Successful defending in soccer—particularly in one vs. one scenarios—is likely to require accurate and rapid foot positioning and anticipation of ball movement, traits that may be associated with an individual’s dribbling performance. In this study, we used metrics of dribbling performance along paths of different curvature to predict defensive performance, where the objective is to steal the ball or prevent being beaten by a single attacker, and contrasts these tests with rankings made by coaches and metrics of the player’s sprinting performance. First, we used generalized linear models to identify the best predictors of individual success in the one vs. one competition based on dribbling speed, sprinting speed and coach ranking. We predicted that both coach rating and dribbling performance would be important predictors of defensive success. Second, we tested how differences in dribbling speed, sprinting speed and coaches’ ranking between two, paired opponents was associated with a defender’s ability to prevent that opponent from scoring against them. We predicted that a greater difference in dribbling performance and coach ranking between opponents would be associated with greater differences in success between paired opponents. Thus, individuals with much better dribbling abilities than their opponent would be able to prevent more goals against them.

## Methods

This research was approved by the University of Queensland Ethics Committee (17-PSYCH-PHD-67-JMC). The form of consent was oral from both the participants and their organisation (soccer club) and all data were analysed anonymously. All players and guardians gave consent at the beginning of the season to all research activities undertaken within their regular training sessions. UQ provided ethical approval for the study with two conditions: (i) verbal consent was obtained from each of the players before the study, and (ii) the football club provided written confirmation that consent was obtained from all player’s and guardians for research at the beginning of the season. We obtained both forms. Twenty-one male players from the Junior Team Football Academy of Londrina State University, in Londrina, Brazil (mean age 17.2 years, SD = 1.1; range 15.4–18.2 years old) participated in the study. This Academy has been used to identify young players for Brazilian professional teams and the National squad since 2001.

The study was conducted within three two-hour sessions as a part of players’ regular training schedule. A one vs. one competition was conducted on days 1 and 3 of the study, while the dribbling and sprinting performances of all players were assessed on day 2. Before testing on any day, all players proceeded through their normal 15 min. warm-up routine with their coach that involved static and dynamic stretching and slow increases in the intensity of running.

### Dribbling performance

The dribbling performance of each player was tested along five different 30 m long paths that varied in curvature. Each path consisted of a 1 m-wide channel, with the outer boundaries marked with 6mm black and yellow plastic chain (Kateli, Brazil). Paths consisted of straight sections interspersed with turns that were always 1m in diameter and either 45° (1/8 of a circle), 90° (1/4 of a circle), 135° (3/8 of a circle) or 180° (1/2 a circle) (S1 Fig). Path 1 had no turns and consisted of a straight 30 m long path. Path 2 had 6 turns, with five 90° turns and one 135° turn, with an average curvature of 0.37 radians.m^-1^. Path 3 had 10 turns, with two 45° turns, two 90° turns and two 135° turns and three 180° turns, with an average curvature of 0.65 radians.m^-1^. Path 4 had 15 turns, with four 45° turns, four 90° turns and two 135° turns, and five 180° turns, with an average curvature of 1.03 radians.m^-1^. Path 5 had 20 turns, with five 45° turns, five 90° turns and four 135° turns, and six 180° turns, with an average curvature of 1.37 radians.m^-1^.

Dribbling speed was quantified by recording the total time taken for an individual to dribble the football through each 30m path. Each player started with the ball behind the starting position and proceeded through the circuit as fast as possible. The data timers counted down from 3 to 1 and then said go, which was used to indicate when the players could start moving and when the data timers would start the stopwatch. Time was stopped when both the player and ball crossed the finish line. Two researchers used stopwatches to record the time taken by a player to complete the circuit and these two times were averaged and taken as a player’s average time over the path. Times were recorded to the nearest 0.01s and if times differed between the two researchers by more than 2% of the total time (fewer than 15 occasions) then they were not included in the analyses. Times were converted to average speed through each path.

Players moved through each test station in groups of 4 or 5 participants, and each group visited all five dribbling performance stations on three separate occasions. Each group of players was randomly assigned a starting station, but all players progressed through stations in the same order: path 1, path 4, path 2, path 3, and path 5. Each player in a group completed the path once at each station before the entire group moved to the next station. Players were asked to complete the path as quickly as possible without the ball going out of the 1m wide pathway. If the ball went outside the path then the test was stopped (defined as a fault) and the individual repeated the trial after a minimum of 2 minutes rest.

We conducted a principal component analysis (PCA) on data of dribbling speed along each of the five paths to reduce dimensionality of the data and extract an overall measure of dribbling speed. The first component of the PCA (PC_D1_) explained 81.9% of the variation observed in the data (S1 Table). All vectors of PC_D1_ loaded in the same direction, and because larger positive values were indicative of higher dribbling speed, PC_D1_ represented a measure of overall dribbling performance.

### Sprinting performance

The sprinting performance of each player was tested along the same five 30 m paths used for the dribbling tests on day 2. After all dribbling tests were completed, players were given a 20 min rest (walking or dynamic stretches) and again rotated through the same paths in groups of 4 to 5. Groups were randomly assigned to a starting station, so that the order of testing was different for each. Every individual within a group completed the path once before the entire group moved on to the next, and each group visited all five performance stations on two separate occasions on the same day. Speeds were measured as described above for dribbling performance.

We conducted a principal component analysis (PCA) on data of sprinting speed along each of the five paths to reduce dimensionality of the data and extract an overall measure of sprinting speed. The first components of the PCA (PC_S1_) explained 72.5% of the variation observed in the data (S1 Table). All vectors of PC_S1_ loaded in the same direction, and because larger positive values were indicative of higher dribbling speed, PC_S1_ represented a measure of overall sprinting performance.

### Coach ranking

We asked the four senior coaches of the Junior Team Football Academy of Londrina State University to rank each player from best (21) to worst (1) in their defensive ability in one vs one contests. All four coaches were asked to discuss this together and come to a consensus and provide one ranking from all coaches combined. Coaches did not watch the dribbling or sprinting assessment of individual players.

### One vs one defensive competition

A one vs one attacking and defending competition was used to replicate situations where an attacker would take on a single defender, which occurs frequently during a game. Competitions were held on a field 15m wide and 25m long, with a 4m x 15m area at one end of the field defined as the scoring zone. Each individual player competed one vs one against 8 to 14 opponents. Each competition between pairs of players consisted of 10 individual bouts, with each player acting as attacker on five occasions and defender on five occasions. This resulted in a total of 1090 individual one vs. one bouts across the 25 individuals. The first player to defend in a pair was chosen at random, and after this, players alternated positioning as attacker and defender. Thus, each player in a pair had the opportunity to defend five times and score up to five points.

At the beginning of each bout, the attacker was positioned behind the line at the opposite end of the field to the scoring zone, with the defender at the center of the field. The objective of the defender was to prevent the attacker from dribbling the ball past them into the scoring zone, using any legal strategy. Each bout ended when either (i) the ball was kicked out of the field or scoring zone (successful defense), (ii) the ball was touched by the attacker in the scoring zone (unsuccessful defense), or (iii) a total time of 30s had elapsed without a result (successful defense).

One vs. one games were conducted in a tournament design [23], so that a hierarchy of best to worst defender could be determined. Individuals were randomly placed into five groups of 5–6 individuals, with all individuals in a group playing each other once. After this first round, all players were then randomly distributed to another five groups of 5–6 individuals, with all individuals playing each other once. These first two rounds were conducted on day 1 of the experiment so that all players competed against 8 unique opponents, providing them with 40 attacking and 40 defending opportunities. Players were allowed to rest for 5–10 minutes when switching between competitors. On day 3, using a smaller number of players (N = 17) we conducted an additional number of one vs. one competitions (N = 4–6) that extended our sample size and allowed us to test the repeatability of defending performance across days.

### Statistical analyses

Correlations among performances were conducted using Pearson’s product moment correlations. We used a false-discovery threshold adjustment to correct for multiple testing of correlations. The effects of dribbling ability, sprinting speed and the coaches’ ranking on the proportion of successful defenses for an individual were analyzed using a binomial, generalized linear model with a logistic link function. PC_D1_, PC_D2_, PCA_S1_, PC_S2_ and coaches’ ranking were included as fixed effects and random intercepts were fitted for each player’s ID. A linear regression model was fit to determine how dribbling ability, sprinting speed and coaches’ ranking affected the average duration of a contest for each defender in the one vs. one competition. A linear mixed effects model was used to determine how relative differences in PC_D1_ (dribbling) and PC_S1_ (sprinting) and the relative differences in coaches’ ranking between paired opponents affected the defender’s success with each specific opponent. Random intercepts were fit for each player’s ID. Where appropriate, normality assumptions were checked with Shapiro—Wilk tests and visual inspections of QQ plots. All analyses were carried out in R 3.4.3 [24]. The data used to perform all statistical analyses is provided within S2 Table.

## Results

### One vs. one defending

In the one vs. one competition, defenders successfully prevented attackers from reaching the scoring zone 62.9 ± 16.4% of the time (mean ± standard deviation). The best performing defender prevented opponents from scoring on 87% of attempts (N = 60 attempts), while the poorest prevented opponents scoring on only 22% of attempts (N = 65 attempts).

Those individuals that successfully defended on day 1 were more likely to successfully defend on day 3 (intra-class correlation coefficient of 0.67). For all individuals, the proportion of successful defending attempts on day 1 was significantly positively correlated with the proportion of successful defences on day 3 (N = 17; r_p_ = 0.49; P = 0.047).

### Determinants of success

**Fig 1:**
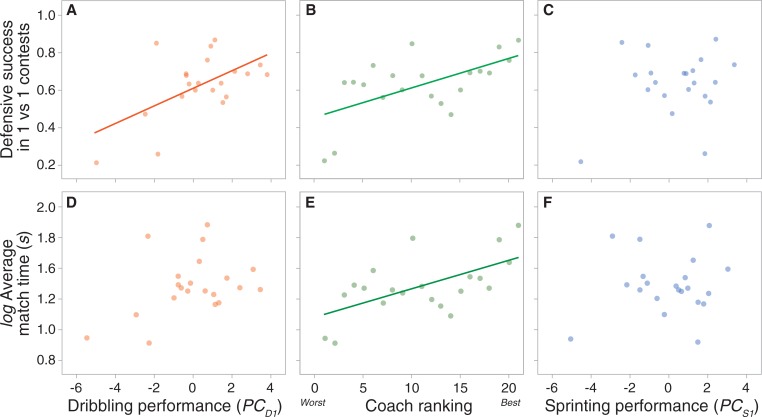
The relationship between a player’s dribbling speed, sprinting speed and coaches’ ranking and their proportion of successfully defended attempts and average length of contests. The proportion of successful defended attempts in the one vs. one competition for each individual was positively associated with their overall metric of dribbling speed (PC_D1_) (N = 21; r = 0.56; P = 0.02) (A) and the coaches ranking of the players (highest number is highest ranked player) (N = 21; r = 0.59; P = 0.02) (B), but not the overall metric of sprint speed (PC_S1_) (C). The average length of a contest for each defender was not significantly correlated with their overall dribbling performance (PC_D1_) (N = 21; r = 0.15; P = 0.61) (D) but was significantly correlated with the ranking (N = 21; r = 0.48; P = 0.044) (E). A player’s overall sprinting performance (PC_S1_) was not significantly associated the average length of a contest (N = 21; r = 0.027; P = 0.91) (F).

A player’s defensive success in the one vs. one competition was significantly positively correlated with their overall dribbling performance (PC_D1_) (N = 21; r = 0.56; P = 0.008) (Fig 1A). A player’s defensive success in the one vs one matches was also significantly positively correlated with each of the four curvy paths (e.g. path curvature of 1.37 radians.m^-1^; N = 21; r = 0.48; P = 0.02) but not the straight path (N = 21; r = 0.39; P = 0.07) (Table 1). Defensive success was also significantly positively correlated with the ranking provided by the coaches (N = 21; r = 0.59; P = 0.004) (Fig 1B).

**Table 1:** Correlations between defensive success of each individual player in the one vs. one competition and measures of sprinting and dribbling performances along each of the five different paths that vary in curvature from 0 to 1.37 radians.m^-1^.

Path curvature (radians.m^-1^)	Dribbling vs Defensive Performance		Sprinting vs Defensive Performance	
	r	P	r	P
0	0.39	0.12	0.33	0.31
0.37	0.59	0.03*	0.11	0.71
0.67	0.53	0.03*	0.18	0.53
1.01	0.54	0.03*	0.46	0.06
1.37	0.48	0.05	0.03	0.89

Statistical significance is denoted by * P<0.05, **P<0.001, and ***P<0.0001.

However, defensive success was only correlated with sprinting speed along the second curviest path (N = 21; r = 0.46; P = 0.03) (Table 1) and was not related to overall sprinting performance (PC_S1_) (N = 21; r = 0.20; P = 0.38) (Fig 1C). Coaches’ rankings were not significantly associated with players’ overall dribbling performance (N = 21; r = 0.27; P = 0.23) or overall sprinting performance (N = 21; r = 0.20; P = 0.38).

Based on the binomial logistic regression model, dribbling performance (PC_D1_) (z = 2.59; P = 0.02) and coaches’ ranking (z = -2.701; P = 0.016) were the only significant predictors of defensive success in the one vs. one competition (S3 Table). Thus, players with higher PC_D1_ values (i.e. better dribbling performance) and better coach rankings were more likely to be successful in the one vs. one competition. The full model that incorporated all five predictors explained 62% of the variation in defensive performance of the players, with approximately half of the variation explained by overall dribbling performance (r^2^ = 0.30) and half by the coaches ranking (r^2^ = 0.37) (S4 Table).

Average match duration for individual players was significantly correlated with their ranking provided by the coaches (N = 21; r = 0.48; P = 0.027) (Fig 1E). Thus, players classified as better defenders by coaches were more likely to have contests that lasted longer than lower-ranked players. However, the average length of time a match lasted was not significantly correlated with a player’s overall dribbling performance (PC_D1_) (N = 21; r = 0.15; P = 0.51) (Fig 1D) or sprinting performance (PC_S1_) (N = 21; r = 0.027; P = 0.91) (Fig 1F). Based on the regression model, only coach ranking (t = -2.211; P = 0.043) was significantly related to average duration of the matches (S5 Table).

**Fig 2:**
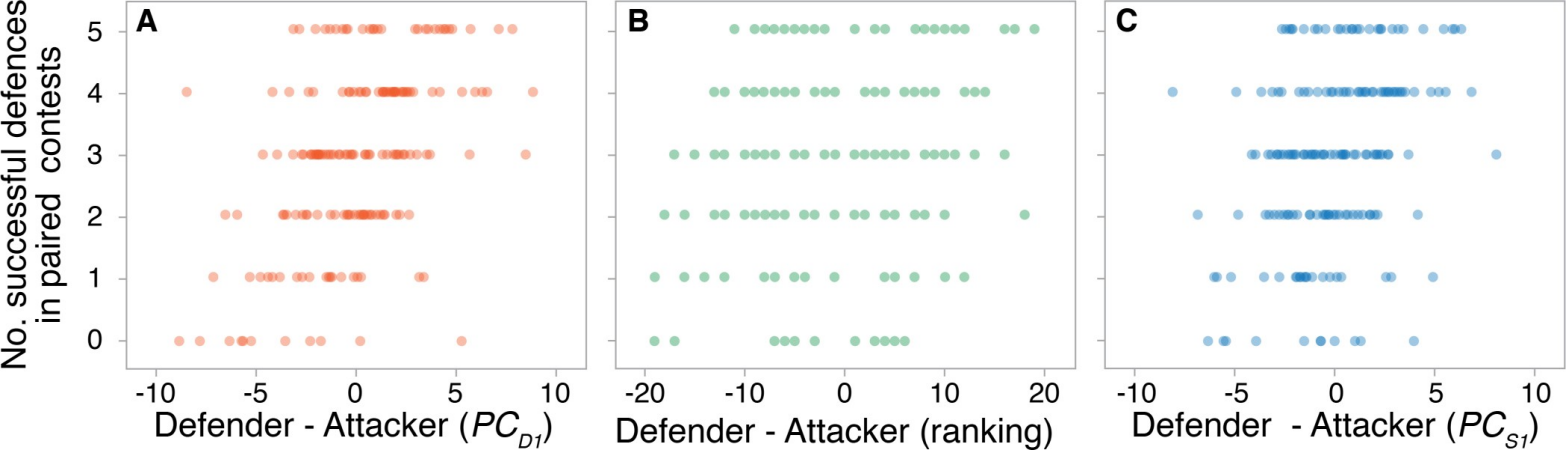
Relationship between the number of goals prevented and relative overall dribbling performance. The number of goals prevented by defenders during each paired competition with an opponent during the one vs. one competition when correlated with the relative overall dribbling performance (defender’s PCD1—attacker’s PC_D1_) (A), the relative coach ranking (defender’s rank- attacker’s rank) (B), and the relative overall sprinting performance (defender’s PC_S1_- attacker’s PC_S1_) (C). Only relative PC_D1_ was a significant predictor of number of goals prevented.

When relative scores were compared between players (i.e. defender minus attacker score), only relative dribbling performance (PC_D1_) predicted defending success (z = 4.19; P < 0.0001) (S6 Table and Fig 2). Thus, defenders with higher dribbling performance compared with their attackers were more likely to prevent a greater number of scores against them.

**Fig 3:**
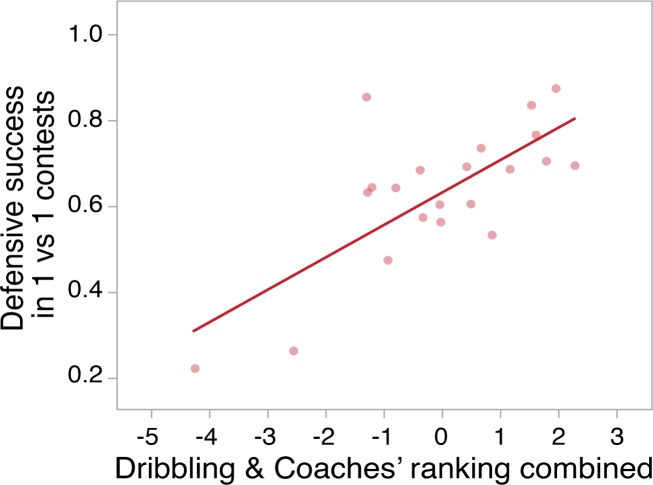
Relationship between defensive success and the combined metric of coach ranking and overall dribbling performance. A player’s defensive success in the one vs. one matches was significantly positively correlated with the combined metric of coach ranking and overall dribbling performance (PC_D1_) (N = 21; r = 0.73; P = 0.0002).

A combined metric of coach ranking and overall dribbling performance (PC_D1_) was calculated by summing the two variables after they were standardized (average of zero and standard deviation of 1). This metric was significantly positively correlated with the player’s defensive success in the one vs. one matches (N = 21; r = 0.73; P = 0.0002) (Fig 3).

## Discussion

The aim of the current study was to show whether dribbling speed, sprinting speed, and coaches’ ranking could be used to predict the defensive performance of soccer players in one vs. one soccer games. Overall, defensive success was best predicted by two factors: the defender’s dribbling performance and the coaches’ ranking, which together explained 62% of the variance in defensive performance. Overall sprinting speed as assessed along the five paths was not significantly related to defensive performance. Relative differences in dribbling performance—but not sprinting or coach ranking—between pairs of opponents were also related to defensive score. In other words, a defender that had a higher relative dribbling performance than their attacker was more likely to prevent the attacker from scoring.

Independently, both dribbling performance and coaches’ ranking explained more than 30% of the variation in defensive success, indicating that coaches are just as proficient at predicting defender success as dribbling performance. Because dribbling performance and coaches’ rankings were not significantly associated, it is likely they identify different attributes important to defensive play. Interestingly, the coaches’ ranking was significantly positively associated with the average time a one vs. one contest lasted. Coaches may rank individuals based on the decision-making ability of the players, so that those players known to be good decision-makers were also those in the one vs. one competition best able to slow down attackers and avoid rushing into tackles. The game-reading skills of soccer players are assumed to underlie successful decision-making behaviour [25, 26]. In contrast, dribbling performance likely reflects another aspect of defence: namely, a defender’s ability to steal the ball from the attacker using rapid and accurate foot movements. Though further work could elucidate the mechanisms driving these differences, the current study demonstrates that including only one metric of closed-skill performance—dribbling performance—doubles the ability of coaches to identify their best defensive players. By summing dribbling performance and the coaches’ ranking provides a simple metric that can be used by coaches to better predict defensive performance in one vs. one contests. In this case, the combined metric of dribbling speed and coach ranking could explain more than 50% of the variance in defensive success in one vs. one competitions. Clearly there is more to defending than simply preventing attackers from getting around them with the ball, so our metric of defensive success may be more applicable to those defenders that are more specialized for preventing the fast, mobile attacking players from beating them.

The current study is of practical value because quantifying the quality of a defender is a major challenge for coaches and talent scouts. High quality defenders can prevent attackers from getting around them, can dispossess attackers, correctly decide when to slow down attackers or pressure them, and can observe the match situation to prevent threatening situations from arising. Thus, quantifying a defender’s quality can be difficult and identifying which traits predict a defender’s ability may be even more problematic. It is possible coaches and talent scouts are judging a defender’s ability to read a game and move into correct positions at the right time. Few, if any, studies have explored which technical traits specifically underlie high quality defending–or even attempted to design closed-skill tests to identify talented defenders. Most previous studies have attempted to explore the possible underlying traits associated with generic success in soccer or elite status [3, 4], not specifically defensive performance.

The one vs. one protocol used here concentrates on a particular component of matches, when defending success can be unambiguously defined and measured. Defenders were asked to prevent attackers from moving past them and scoring and to dispossess the attacker if possible. A closed-skill measure of individual dribbling performance, as presented here, may capture a player’s capacity for accurate and rapid foot positioning, dynamic balance, and anticipation of ball movement, all of which could affect a defender’s ability to dispossess their opponent. In the current study, dribbling speed rather than sprint speed predicted defensive performance, yet it is possible that another, untested metric of technical ability, such as kicking accuracy or juggling, would also be an equally good predictor of defensive performance. Future studies should also explore how dribbling performance is associated with defensive performance in both small-sided games and 11 v 11 matches where defending occurs against multiple agents. We would expect that predictors of defensive ability would differ with the quality of the opposition and type and structure of the game being played. A more holistic understanding of what predicts defensive ability in 11 v 11 matches will require further experimentation.

Scientific talent identification programs in most team sports, like soccer, will be largely unsuccessful until it is recognized that there is no single pathway to success. Comparisons of technical and physical traits between elite and non-elite groups may elucidate the minimal levels required to play elite standards, but they will not allow one to identify the traits associated with success within elite competitions. Soccer is a complex team sport with clear divisions of labour among individual team members, so that there is specialization to different tasks across and even within positions (e.g. defenders, attackers)[27]. Success in each role within a team will be defined by the different combination or set of traits that is best suited to the specific tasks they must perform in a match. Thus, it is unrealistic to expect success to be defined by a single trait and, in fact, we should expect elite and sub-elite groups to substantially overlap in their performances in any single trait.

To better identify players that will be successful within elite competitions, the combinations of traits associated with success must be discovered across different positions and tasks within a team. These studies will require large sample sizes that encompass the natural variation in combinations of trait and should consider the position or role of the player—for example, certain combinations of traits may be associated with high success for defenders but not for attackers. Analytical frameworks for these analyses already exist in evolutionary biology [28–30], where they are used to show how multi-dimensional trait combinations are associated with “success” (i.e. offspring or survival). Reframing these analyses to understand “success” in sport could revolutionise approaches to talent identification in team sports that have clear divisions of labour between roles within a team.

## Supporting information

S1 Fig The five 30 m-long paths used to assess dribbling and sprinting performance for the individual players in our study. The curvature of each path was (A) 0 radians.m^-1^, (B) 0.37 radians.m^-1^, (C) 0.67 radians.m^-1^, (D) 1.03 radians.m^-1^, and (E) 1.37 radians.m^-1^. The outline of each path was marked with 6 mm plastic chain and the width of each path was 1m, and any 45° (1/8 of a circle), 90° (1/4 of a circle), 135° (3/8 of a circle) or 180° (1/2 a circle) turns had a radius of 1m. The number of 0.5 m long sections (grey) along each path shows the lengths between each turn, with a 90° turn represented in green and 45° in yellow.
(DOCX)

S1 Table Principal components analysis matrix of dribbling and sprinting performance (N = 21) along the five paths that differ in curvature from 0 to 1.37 radians.m^-1^. Factor loadings of each measured variable and the direction in which they contribute towards the components are shown. See the text for a description of each trait. The first component of the PCA on dribbling performance (PC_D1_) explained 81.9% of the variation in the data and the second component (PC_D2_) explained 11.4% of the variation. The first component of the PCA on sprinting performance (PC_S1_) explained 72.5% of the variation in the data and the second component (PC_S2_) explained 14.6% of the variation.
(DOCX)

S2 Table Defensive performance data set. Summary of the data set used to assess defensive performance of individual players in one vs. one soccer games.
(DOCX)

S3 Table Summary results from the binomial logistic regression, testing the effects of dribbling ability, sprinting speed and coach ranking on the defensive success of each player in the one vs. one competition. (DOCX)

S4 Table Proportion of variance explained by each fixed effect in the binomial logistic regression testing the effects of dribbling ability, sprinting speed and coach ranking on the defensive success of each player in the one vs. one competition. (DOCX)

S5 Table Summary results from the linear regression, testing the effects of dribbling ability, sprinting speed and coach ranking on the average length of a contest for each defender in the one vs. one competition. (DOCX)

S6 Table Summary results from the linear mixed effects model, testing the effects of relative dribbling ability (PC_D1_), relative sprinting speed (PC_S1_) and relative coach ranking on the defender’s success in each paired bout in the one vs. one competition. (DOCX)
