# Supplementary material for: Predicting the defensive performance of individual players in one vs. one soccer games
Source: PLoS One. 2018 Dec 31;13(12):e0209822. doi: 10.1371/journal.pone.0209822 (PMC6312280; doi:10.1371/journal.pone.0209822)
Supplement: S1 Fig — The curvature of each path was (A) 0 radians.m-1, (B) 0.37 radians.m-1, (C) 0.67 radians.m-1, (D) 1.03 radians.m-1, and (E) 1.37 radians.m-1. The outline of each path was marked with 6 mm plastic chain and the width of each path was 1m, and any 45° (1/8 of a circle), 90° (1/4 of a circle), 135° (3/8 of a circle) or 180° (1/2 a circle) turns had a radius of 1m. The number of 0.5 m long sections (grey) along each path shows the lengths between each turn, with a 90° turn represented in green and 45° in yellow. (DOCX) [file pone.0209822.s001.docx]

**Supporting Information**

**S1 Fig. The five 30 m-long paths used to assess dribbling and sprinting performance for the individual players in our study.** The curvature of each path was (A) 0 radians.m^-1^, (B) 0.37 radians.m^-1^, (C) 0.67 radians.m^-1^, (D) 1.03 radians.m^-1^, and (E) 1.37 radians.m^-1^. The outline of each path was marked with 6 mm plastic chain and the width of each path was 1m, and any 45**°** (1/8 of a circle), 90**°** (1/4 of a circle), 135**°** (3/8 of a circle) or 180**°** (1/2 a circle) turns had a radius of 1m. The number of 0.5 m long sections (grey) along each path shows the lengths between each turn, with a 90° turn represented in green and 45° in yellow.
