# Supplementary material for: Predicting the defensive performance of individual players in one vs. one soccer games
Source: PLoS One. 2018 Dec 31;13(12):e0209822. doi: 10.1371/journal.pone.0209822 (PMC6312280; doi:10.1371/journal.pone.0209822)
Supplement: S1 Table — Factor loadings of each measured variable and the direction in which they contribute towards the components are shown. See the text for a description of each trait. The first component of the PCA on dribbling performance (PCD1) explained 81.9% of the variation in the data and the second component (PCD2) explained 11.4% of the variation. The first component of the PCA on sprinting performance (PCS1) explained 72.5% of the variation in the data and the second component (PCS2) explained 14.6% of the variation. (DOCX) [file pone.0209822.s002.docx]

**S1 Table.** **Principal components analysis matrix of dribbling and sprinting performance (N = 21) along the five paths that differ in curvature from 0 to 1.37 radians.m^-1^.** Factor loadings of each measured variable and the direction in which they contribute towards the components are shown. See the text for a description of each trait. The first component of the PCA on dribbling performance (PCA_D1_) explained 81.9% of the variation in the data and the second component (PCA_D2_) explained 11.4% of the variation. The first component of the PCA on sprinting performance (PCA_S1_) explained 72.5% of the variation in the data and the second component (PCA_S2_) explained 14.6% of the variation.

| **Path curvature**  **(radians.m^-1^)** | **Dribbling Performance** | | **Sprinting**  **Performance** | |
| --- | --- | --- | --- | --- |
|  | **PC_D1_** | **PC_D2_** | **PC_S1_** | **PC_S2_** |
| 0 | 0.74 | 0.66 | 0.75 | 0.58 |
| 0.37 | 0.91 | -0.25 | 0.95 | 0.01 |
| 0.67 | 0.96 | -0.12 | 0.92 | -0.17 |
| 1.01 | 0.95 | -0.21 | 0.75 | -0.58 |
| 1.37 | 0.94 | 0.05 | 0.87 | 0.18 |
| Total variance | 81.9 | 11.4 | 72.5 | 14.6 |
