# Supplementary material for: Predicting the defensive performance of individual players in one vs. one soccer games
Source: PLoS One. 2018 Dec 31;13(12):e0209822. doi: 10.1371/journal.pone.0209822 (PMC6312280; doi:10.1371/journal.pone.0209822)
Supplement: S2 Table — Summary of the data set used to assess defensive performance of individual players in one vs. one soccer games. (DOCX) [file pone.0209822.s003.docx]

**S2 Table. Defensive performance data set.** Summary of the data set used to carry out statistical analyses.

|  | % |  |  |  |  | No. |  |  | Average |
| --- | --- | --- | --- | --- | --- | --- | --- | --- | --- |
| Player | successful | PC1 |  |  |  | successful | Defensive | Coaches' | time |
| ID | defenses | dribble | PC 2 dribble | PC 1 sprint | PC 2 sprint | defenses | trials | ranking | defending |
| 1 | 0.87 | 0.723705 | -0.046683697 | 2.0733537 | 0.8414842 | 52 | 60 | 1 | 9.363636364 |
| 2 | 0.6 | 0.6096495 | 0.106166311 | 0.6338757 | 0.6713238 | 33 | 55 | 13 | 8.727272727 |
| 3 | 0.73 | 3.0770788 | 0.027493551 | 3.0376825 | -0.6363926 | 44 | 60 | 16 | 8.75 |
| 4 | 0.26 | -2.2349721 | 0.896544604 | 1.4895519 | 0.416831 | 13 | 50 | 20 | 6.5 |
| 5 | 0.64 | -0.1388692 | 0.129131586 | -1.1145606 | -0.5037633 | 35 | 55 | 18 | 9.090909091 |
| 6 | 0.64 | 1.0592771 | -1.18133139 | 2.0276729 | 0.2974216 | 35 | 55 | 19 | 7.545454545 |
| 7 | 0.69 | -0.7602573 | -0.508912726 | -1.3433125 | -1.2901477 | 24 | 35 | 6 | 9 |
| 8 | 0.68 | 3.4363924 | -0.648498665 | 0.4830806 | 0.3241478 | 41 | 60 | 14 | 7.25 |
| 9 | 0.22 | -5.4108898 | -0.592153886 | -5.0703983 | 1.0381345 | 14 | 65 | 21 | 7.384615385 |
| 10 | 0.83 | 0.5163059 | -0.021575328 | -1.4968296 | -1.2010125 | 10 | 12 | 3 | 9.5 |
| 11 | 0.7 | 1.733684 | 0.445890955 | 0.8427278 | 0.1605783 | 42 | 60 | 5 | 8.333333333 |
| 12 | 0.56 | 1.3052104 | 1.164183186 | 1.4967425 | 0.2930216 | 31 | 55 | 15 | 7.909090909 |
| 13 | 0.6 | -0.2751389 | -1.895318649 | -1.4985104 | -2.3846434 | 21 | 35 | 7 | 8.857142857 |
| 14 | 0.68 | -0.745638 | -0.424930791 | -2.1797054 | 0.1135697 | 44 | 65 | 11 | 7.846153846 |
| 15 | 0.63 | -0.616787 | -0.007380342 | 0.9450191 | -0.3662762 | 38 | 60 | 17 | 8.666666667 |
| 16 | 0.76 | 0.3347617 | 0.976390296 | 1.2677887 | -0.3286747 | 44 | 58 | 2 | 9 |
| 17 | 0.85 | -2.3024336 | 0.036979269 | -2.8958659 | 0.9609895 | 34 | 40 | 12 | 8.75 |
| 18 | 0.57 | -0.9704872 | -0.324804251 | -0.6382396 | 0.1624938 | 34 | 60 | 10 | 8.333333333 |
| 19 | 0.69 | 2.4113534 | 0.814687664 | 0.386925 | 0.3832661 | 24 | 35 | 4 | 7.285714286 |
| 20 | 0.47 | -2.8888966 | 1.254189415 | -0.2408805 | 1.3387864 | 26 | 55 | 8 | 7.636363636 |
| 21 | 0.53 | 1.1369516 | -0.200067112 | 1.7938824 | -0.2911379 | 32 | 60 | 9 | 8.166666667 |
