# Supplementary material for: Predicting the defensive performance of individual players in one vs. one soccer games
Source: PLoS One. 2018 Dec 31;13(12):e0209822. doi: 10.1371/journal.pone.0209822 (PMC6312280; doi:10.1371/journal.pone.0209822)
Supplement: S3 Table — (DOCX) [file pone.0209822.s004.docx]

**S3 Table. Summary results from the binomial logistic regression, testing the effects of dribbling ability, sprinting speed and coach ranking on the defensive success of each player in the one vs. one competition.**

| Path (radians.m^-1^) | Estimate | Std. Error | z value | Pr(>\|z\|) |
| --- | --- | --- | --- | --- |
| Intercept | 1.493 | 0.394 | 3.793 | 0.018* |
| PC_D1_ | 0.219 | 0.084 | 2.59 | 0.021* |
| PC_D2_ | -0.069 | 0.189 | -0.362 | 0.722 |
| PC_S1_ | -0.076 | 0.094 | -0.805 | 0.434 |
| PC_S2_ | 0.005 | 0.174 | 0.029 | 0.978 |
| Coach rating | -0.447 | 0.166 | -2.701 | 0.016* |
