# Supplementary material for: Predicting the defensive performance of individual players in one vs. one soccer games
Source: PLoS One. 2018 Dec 31;13(12):e0209822. doi: 10.1371/journal.pone.0209822 (PMC6312280; doi:10.1371/journal.pone.0209822)
Supplement: S5 Table — (DOCX) [file pone.0209822.s006.docx]

**S5 Table. Summary results from the linear regression, testing the effects of dribbling ability, sprinting speed and coach ranking on the average length of a contest for each defender in the one vs. one competition.**

| Path (radians.m^-1^) | Estimate | Std. Error | z value | Pr(>\|z\|) |
| --- | --- | --- | --- | --- |
| Intercept | 9.248 | 0.463 | 19.967 | 3.23e-12*** |
| PC_D1_ | -0.005 | 0.115 | -0.041 | 0.968 |
| PC_D2_ | -0.015 | 0.254 | -0.059 | 0.954 |
| PC_S1_ | -0.034 | 0.122 | -0.277 | 0.786 |
| PC_S2_ | -0.382 | 0.216 | -1.767 | 0.098 |
| Coach rating | -0.448 | 0.202 | -2.211 | 0.043* |
