# Supplementary material for: Predicting the defensive performance of individual players in one vs. one soccer games
Source: PLoS One. 2018 Dec 31;13(12):e0209822. doi: 10.1371/journal.pone.0209822 (PMC6312280; doi:10.1371/journal.pone.0209822)
Supplement: S6 Table — (DOCX) [file pone.0209822.s007.docx]

**S6 Table. Summary results from the linear mixed effects model, testing the effects of relative dribbling ability (PC_D1_), relative sprinting speed (PC_S1_) and relative coach ranking on the defender’s success in each paired bout in the one vs. one competition.**

| Path (radians.m^-1^) | Estimate | Std. Error | Tvalue | Pr(>\|z\|) |
| --- | --- | --- | --- | --- |
| Intercept | 2.944 | 0.128 | 23. 064 | 4.09e-14*** |
| PC_D1_ | 0.204 | 0.049 | 4.191 | 5.95e-05*** |
| PC_S1_ | -0.0003 | 0.053 | -0.007 | 0.995 |
| Coach ranking | 0.016 | 0.013 | 1.290 | 0.200 |
